# MiR-486-3p was downregulated at microRNA profiling of adrenals of multiple endocrine neoplasia type 1 mice, and inhibited human adrenocortical carcinoma cell lines

**DOI:** 10.1038/s41598-021-94154-z

**Published:** 2021-07-20

**Authors:** Su-Chen Li, Azita Monazzam, Masoud Razmara, Xia Chu, Peter Stålberg, Britt Skogseid

**Affiliations:** 1 Department of Medical Sciences, Uppsala University, 751 85 Uppsala, Sweden; 2 Department of Surgical Sciences, Uppsala University, Uppsala, Sweden; 3 Science for Life Laboratory in Uppsala, Uppsala, Sweden

**Keywords:** Cancer, Molecular biology

## Abstract

Adrenocortical carcinoma is a rare aggressive disease commonly recurring regardless of radical surgery. Although data on genomic alterations in malignant tumors are accumulating, knowledge of molecular events of importance for initiation of adrenocortical transformation is scarce. In an attempt to recognize early molecular alterations, we used adrenals from young multiple endocrine neoplasia type 1 conventional knock-out mice (*Men1*^+/−^) closely mimicking the human MEN1 trait (i.e. transformation of pituitary, parathyroid, endocrine pancreatic, and adrenocortical cells). MicroRNA array and hierarchical clustering showed a distinct pattern. Twenty miRNAs were significantly upregulated and eleven were downregulated in *Men1*^+/−^ compared to wild type littermates. The latter included the known suppressor miRNA miR-486-3p, which was chosen for transfection in human adrenocortical carcinoma cell lines H295R and SW13. Cell growth decreased in miR-486-3p overexpressing clones and levels of the predicted target gene fatty acid synthase (FASN) and its downstream product, palmitic acid, were lowered. In conclusion, heterozygous inactivation of *Men1* in adrenals results in distinct miRNA profile regulating expression of genes with impact on tumorigenesis, e.g. transcription, nucleic acid and lipid metabolism. Low levels of miR-486-3p in the early stages of transformation may contribute to proliferation by increasing FASN and thus fatty acid production. FASN as a potentially druggable target for treatment of the devastating disease adrenocortical carcinoma warrants further studies.

## Introduction

The rare disease multiple endocrine neoplasia type 1 (MEN1) is an autosomal dominantly inherited trait. Classically MEN 1 patients suffer from tumors in the pituitary, the parathyroid glands, and the endocrine pancreas^1^. More than one third of gene carriers also develop enlargements of the adrenals^2^, which frequently represent bilateral cortical hyperplasia without hormone overproduction. These proliferating adrenals may progress into adenomas and in rare cases even adrenocortical carcinomas^2,3^. Since more than a decade both conventional and conditional *Men1* knock-out mice models, closely mimicking the human trait, have been made available for researchers^4,5^.

**Figure 1:**
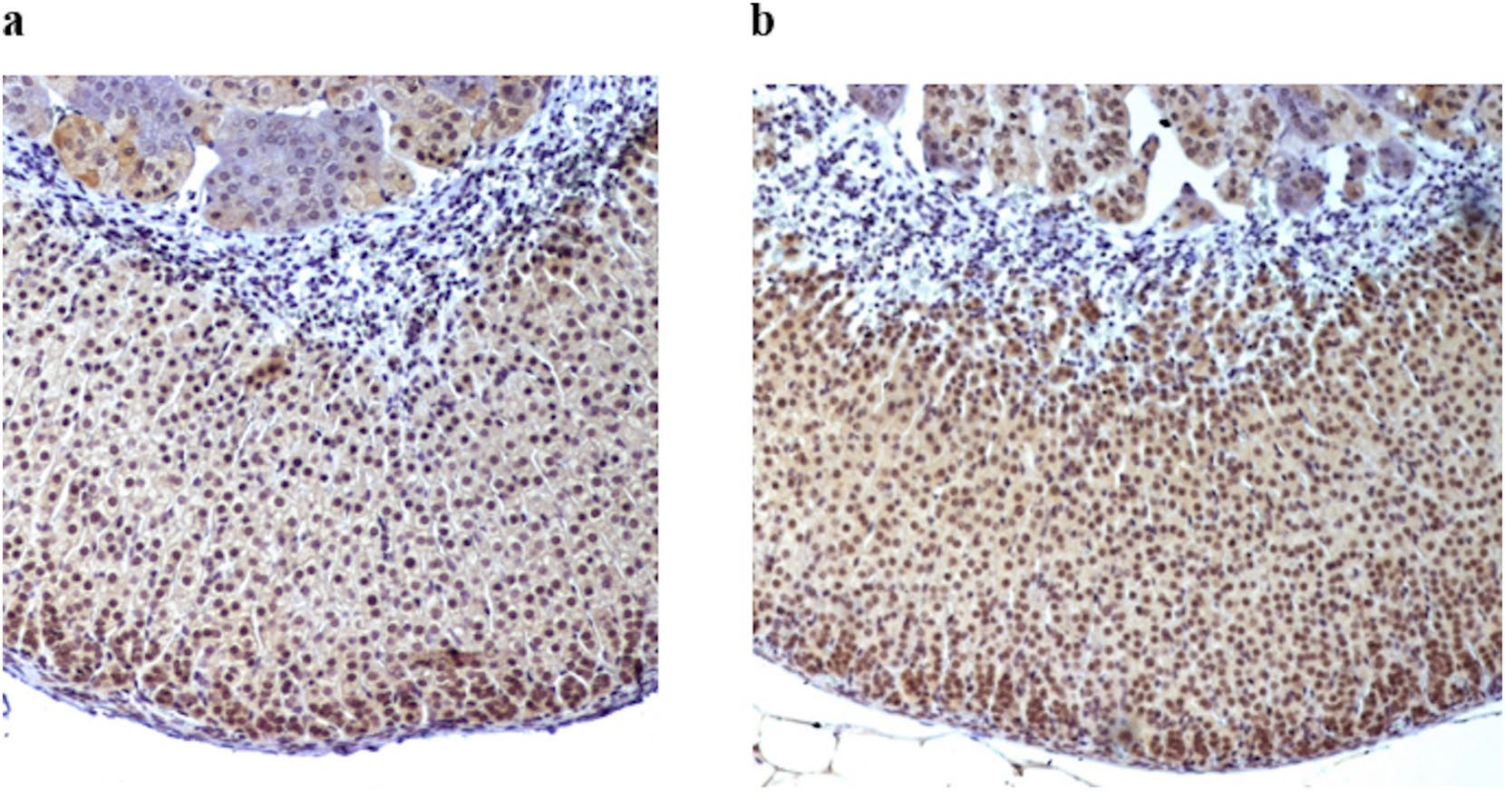
Menin immunoreactivity in (**a**) *Men1*^+/−^ and (**b**) *Men1*^+/+^ adrenal tissue.

The benign, yet proliferating, adrenals of both MEN1 patients and mice retain the wild type allele of the gene^2^, and the protein menin^6^ is expressed to what seems to be the same level and subcellular localization as in the adrenals of humans and wild type mice (Fig. 1). Thus inactivation of a single allele in gene carriers is obviously enough to initiate increased proliferation of the cells of the adrenal cortex, which by definition implies that *MEN1* is a haploinsufficient suppressor in these cells^7^. Loss of heterozygosity of MEN1 is frequently seen in sporadic adrenocortical carcinoma and MEN1 has been considered a driver gene in these tumors^8–11^. In addition, studies of pancreatic islets from heterozygous *Men1* mice and human duodenal neuroendocrine tumors from MEN1 patients have shown that inactivation of a single allele may be sufficient for initiating tumorigenesis also in these cells^12,13^.

The understanding of the molecular events of importance for adrenocortical transformation into sporadic aggressive carcinomas is increasing. Substantial efforts have indeed provided interesting data, e*.g.* whole exome sequencing^14^, proteomic profiling^15^ and microRNA array^16^. However, molecular events responsible for initiating early adrenal tumorigenesis are vastly unknown.

MicroRNAs (miRNAs) are endogenous, short, non-coding RNAs that are post-transcriptional regulators and regulate specific genes by binding to their target genes in the 3’ untranslated region resulting in either translational repression or mRNA degradation^17^. These short sequences control about 50% of the entire human genome^18^. Altered expression patterns of post-transcriptional regulators such as miRNAs are frequent findings in studies of transformation. It has been reported that miRNAs might act either as oncogenes or tumor suppressors during tumorigenesis and progression^19,20^. They are also important regulators of complex gene networks involved in a variety of biological processes, such as cell proliferation, differentiation, apoptosis, development and metabolism^21–23^. As an attempt to recognize potential miRNAs of importance for onset of proliferation in adrenocortical cells, we performed Affymetrix miRNA array from adrenal glands of conventional *Men1* heterozygous knock-out mice (*Men1*^+/−^). To enable recognition of early molecular events we chose to study the adrenals of relatively young adult mice, i.e. 10 months old, possibly before long-term proliferation results in further molecular alterations un-related to constitutional heterozygous *Men1* inactivation. Moreover, we used cell lines to study whether one of the differentially regulated miRNAs and its target genes might be of relevance also in progression of human adrenocortical carcinoma.

## Results

### Differentially expressed miRNAs

**Figure 2:**
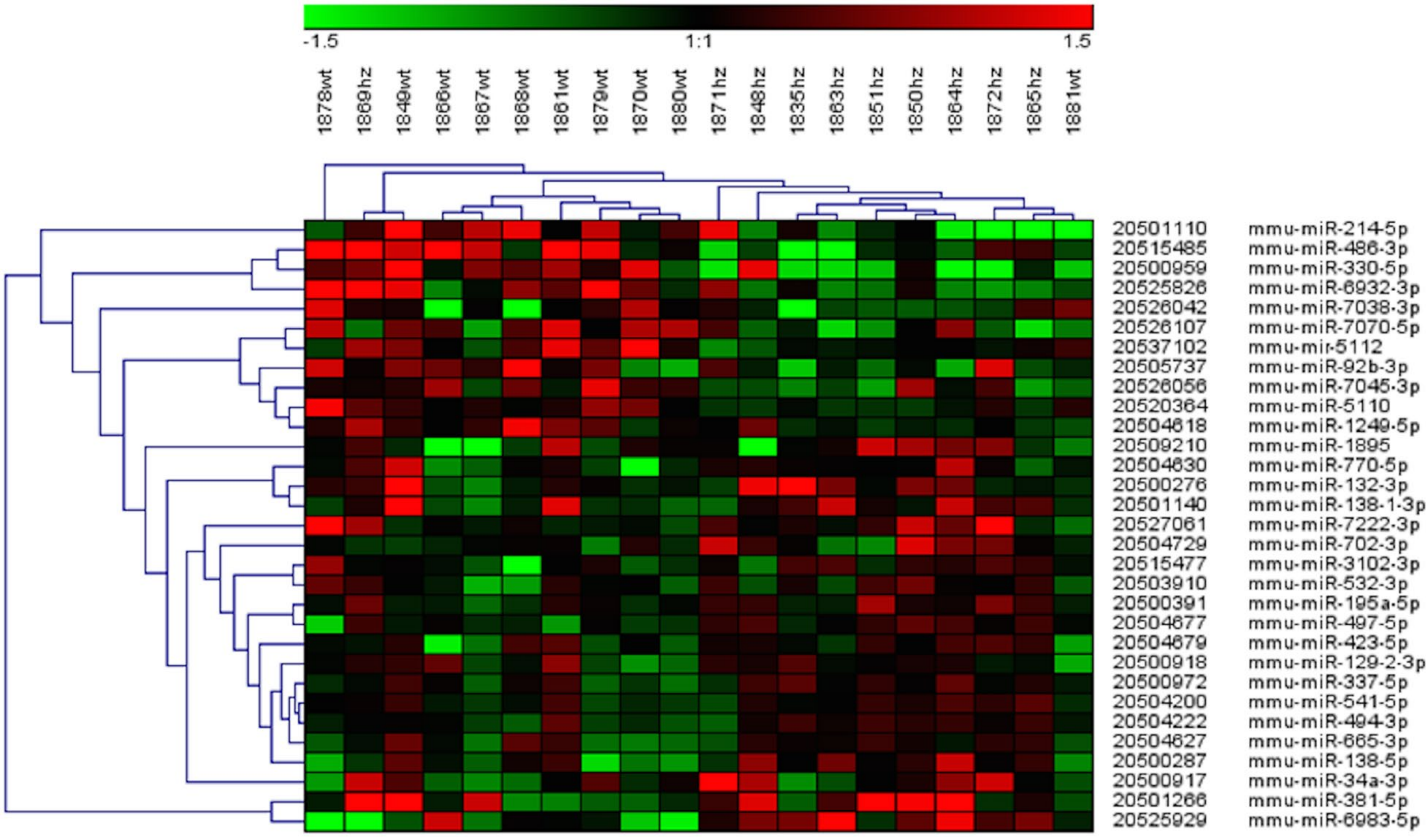
Bidirectional hierarchical clustering and heat map of the 31 significantly differentially expressed miRNAs using software Genesis.

The miRNA array profiling, performed on RNA prepared from the adrenal glands of ten *Men1*^+/−^ and ten wild type (*Men*^+/+^) mice revealed a distinct expression pattern. A hierarchical cluster analysis showed that nine out of ten *Men1*^+/−^ mice were in the same cluster and also nine out of ten *Men1*^+/+^ mice were grouped together (Fig. 2). Eleven miRNAs were significantly downregulated and 20 were significantly upregulated in *Men1*^+/−^ compared to *Men1*^+/+^ (*P* < 0.05) (Table 1). The ratios of up- or down- regulation among significantly differentially expressed miRNAs ranged from 20 to 106%, mean ratio 42.3%. Seven of the differentially regulated miRNAs have earlier been reported as either suppressor or oncogene miRNAs. Among the eleven downregulated miRNAs in *Men1*^+/−^ three were known tumor suppressors (miR-486-3p, miR-330-5p and miR-214-5p) and these three were most highly ranked according to fold change (−1.33, −1.14 and −1.05, respectively). On the other hand, suppressor miR-497-5p and miR-195a-5p were significantly upregulated. Oncogenes miRNAs miR-494-3p and miR-132-3p were significantly enhanced in *Men1*^+/−^.

**Table 1:** Significantly differentially expressed miRNAs in adrenals of *Men1*^+/-^ compared to Men1^+/+^, ranked according to fold change.

miRNAs ID	log2 Fold change (Men1^+/-^/Men1^+/+^)	*p*-value	Regulation	% (*Men1*^+/−^ vs. *Men1*^+/+^)
mmu-miR-486-3p	−1.33	< 0.05	Down	−60
mmu-miR-330-5p	−1.14	< 0.05	Down	−55
mmu-miR-214-5p	−1.05	< 0.05	Down	−52
mmu-miR-7070-5p	−0.93	< 0.05	Down	−48
mmu-miR-6932-3p	−0.80	< 0.05	Down	−42
mmu-miR-92b-3p	−0.61	< 0.05	Down	−35
mmu-miR-7045-3p	−0.55	< 0.05	Down	−32
mmu-mir-5112	−0.48	< 0.01	Down	−28
mmu-miR-7038-3p	−0.44	< 0.05	Down	−26
mmu-miR-5110	−0.43	< 0.001	Down	−26
mmu-miR-1249-5p	−0.30	< 0.05	Down	−20
mmu-miR-541-5p	0.32	< 0.01	Up	25
mmu-miR-3102-3p	0.32	< 0.05	Up	25
mmu-miR-770-5p	0.33	< 0.01	Up	25
mmu-miR-337-5p	0.33	< 0.05	Up	26
mmu-miR-129-2-3p	0.34	< 0.05	Up	27
mmu-miR-532-3p	0.37	< 0.05	Up	29
mmu-miR-423-5p	0.38	< 0.05	Up	30
mmu-miR-494-3p	0.39	< 0.01	Up	31
mmu-miR-702-3p	0.41	< 0.05	Up	33
mmu-miR-665-3p	0.41	< 0.05	Up	33
mmu-miR-195a-5p	0.44	< 0.001	Up	36
mmu-miR-132-3p	0.48	< 0.05	Up	40
mmu-miR-497-5p	0.48	< 0.001	Up	40
mmu-miR-7222-3p	0.53	< 0.01	Up	45
mmu-miR-138–1-3p	0.58	< 0.001	Up	49
mmu-miR-1895	0.66	< 0.05	Up	58
mmu-miR-138-5p	0.70	< 0.05	Up	63
mmu-miR-34a-3p	0.75	< 0.05	Up	68
mmu-miR-381-5p	0.98	< 0.05	Up	98
mmu-miR-6983-5p	1.04	< 0.001	Up	106

### Selection of MiR-486-3p and six potential target genes

We selected the suppressor miR-486-3p for further investigation because it was top ranked among downregulated miRNAs in *Men1*^+/−^, showing a foldchange of -1.33 (Table 1). In addition, among the multitude of predicted potential miR-486-3p target genes altogether six genes (*ALDH2*, *FASN*, *GDI1*, *HINT1*, *KCND3* and *MDGA1*) (Table 2) corresponded to results from proteomics profiling of adrenals of the same *Men1* knock-out strain^24^. Increased expression of one or more of these proteins, as a result of decreased expression of miR-486-3p, may thus be of relevance for adrenocortical transformation. This hypothesis was further tested in expression studies in human adrenocortical cell lines (below).

**Table 2:** Predicted miR-486-3p target genes identified by TargetScan.

Symbol	Description	Biological function
*ALDH2*	Aldehyde dehydrogenase 2	Metabolic process
*FASN*	Fatty acid synthase	Fatty acid metabolic process
*GDI1*	GDP dissociation inhibitor 1	GTPase mediated signal transduction
*HINT1*	Histidine triad nucleotide binding protein 1	Apoptotic process
*KCND3*	Potassium voltage-gated channel subfamily D member 3	Ion transport
*MDGA1*	MAM domain containing glycosylphosphatidylinositol	Nervous system development

### Overexpression of miR-486-3p in adrenocortical carcinoma cell lines inhibited proliferation

**Figure 3:**
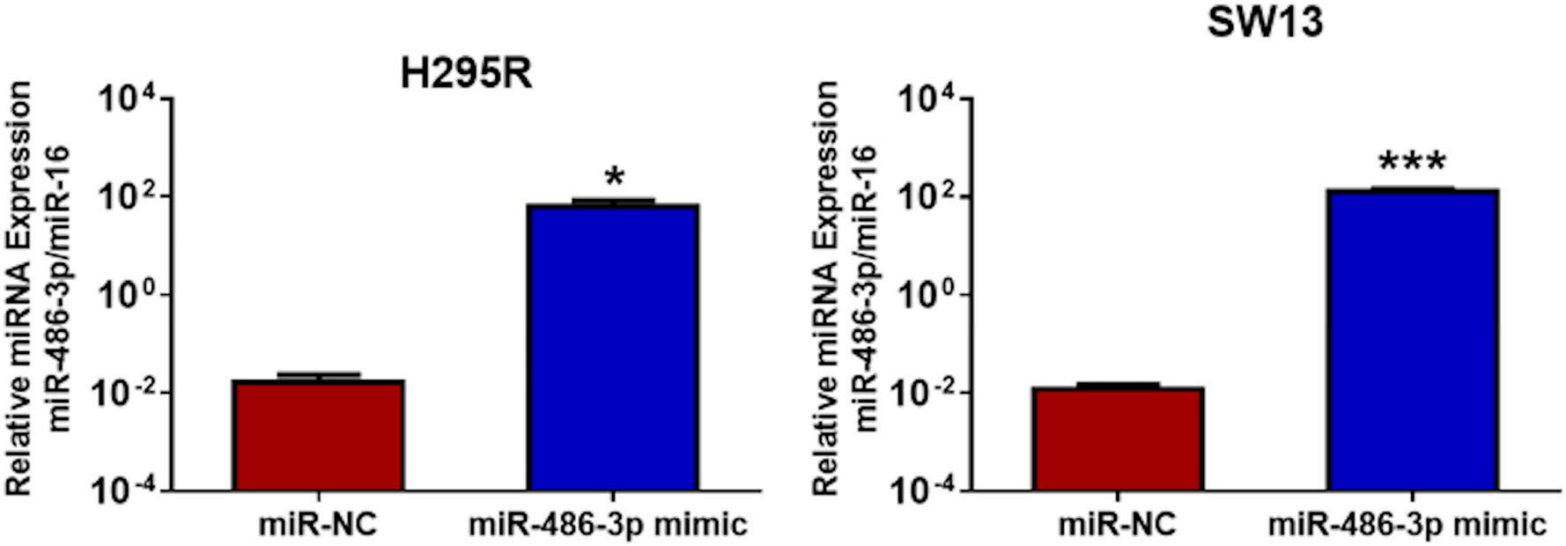
QRT-PCR analysis comparing the expression of miR-486-3p in H295R and SW13 cells, transfected either with miR-486-3p mimic or with scrambled miRNA mimic (miR-NC). The experiments were performed three times.

**Figure 4:**
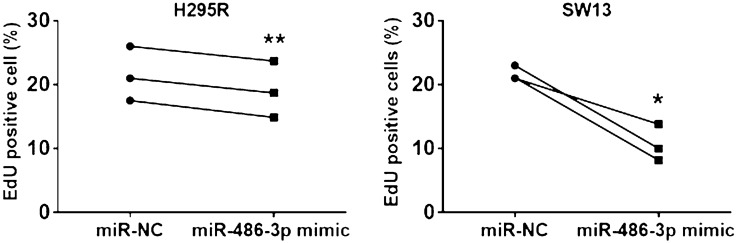
Cell proliferation analysis using EdU flow-cytometry assay. H295R and SW13 cells were transfected with miR-486-3p mimic and miR-NC (control cells) for two days. EdU incorporation was significantly inhibited in H295R miR-486-3p mimic cells (*p* < 0.01) and SW13 miR-486-3p mimic cells (*p* < 0.05) compared to miR-NC cells. The experiments were performed three times.

The established human adrenocortical carcinoma cell lines H295R and SW13 were transfected with miR-486-3p mimics in order to recognize potential biological functions of miR-486-3p in human adrenocortical transformation. The expression level of miR-486-3p after transfection, quantified by QRT-PCR, increased about 10,000-fold in both cell lines compared to miRNA negative control (miR-NC) cells (Fig. 3). Potential effects on proliferation and cell growth was assessed by performing EdU flow-cytometry assay. The assay showed that approximately 22% of miR-NC cells of both cell lines incorporated EdU, whereas the miR-486-3p overexpressing cells incorporated EdU to significantly lower degree indicative of decreased proliferation (Fig. 4). MiR-486-3p overexpressing H295R revealed decreased EdU incorporation by mean 11.55% ± 1.76 (SEM), from mean 21.50% ± 2.47 (SEM) EdU positive cells to mean 19.10% ± 2.55 (SEM) (*p* < 0.01). Corresponding measurements for SW13 showed that miR-486-3p overexpressing clones decreased EdU incorporation by mean 50.66% ± 8.30 (SEM) of levels in controls, from 21.7% ± 0.65 (SEM) to 10.65% ± 1.17 (SEM) EdU positive cells (*p* < 0.05).

### MiR-486-3p regulated FASN expression in H295R and SW13 cells

**Figure 5:**
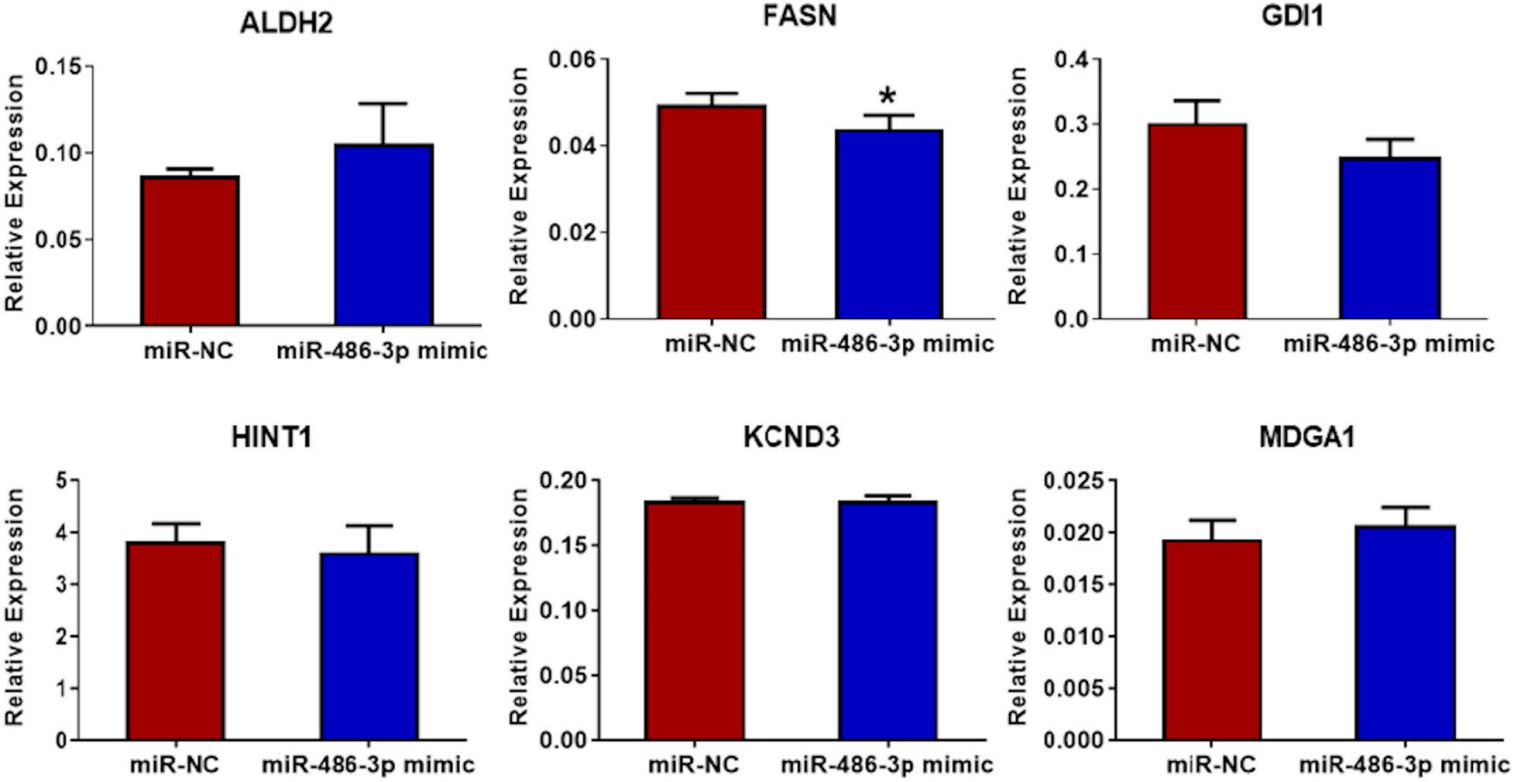
QRT-PCR analysis of potential targets of miR-486-3p in H295R cells**.** Among the six selected potential targets, FASN was the only gene showing significant downregulation (*p* < 0.05) in miR-486-3p mimic transfected H295R cells compared to miR-NC cells. The experiments were performed three times.

**Figure 6:**
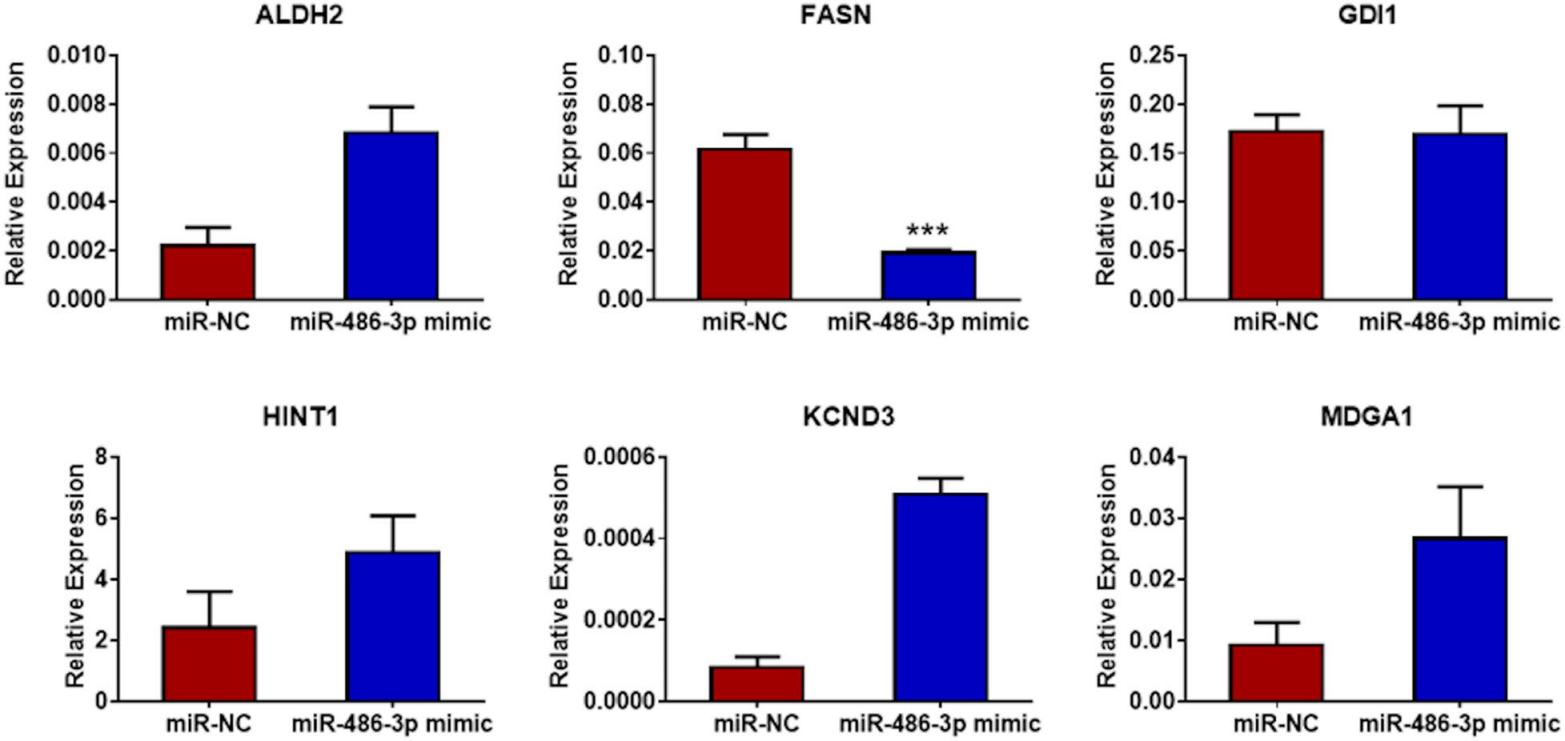
QRT-PCR analysis of potential targets of miR-486-3p in SW13 cells. MiR-486-3p mimic transfected SW13 cells revealed significantly downregulated levels of FASN (*p* < 0.001) compared to miR-NC. The experiments were performed three times.

**Figure 7:**
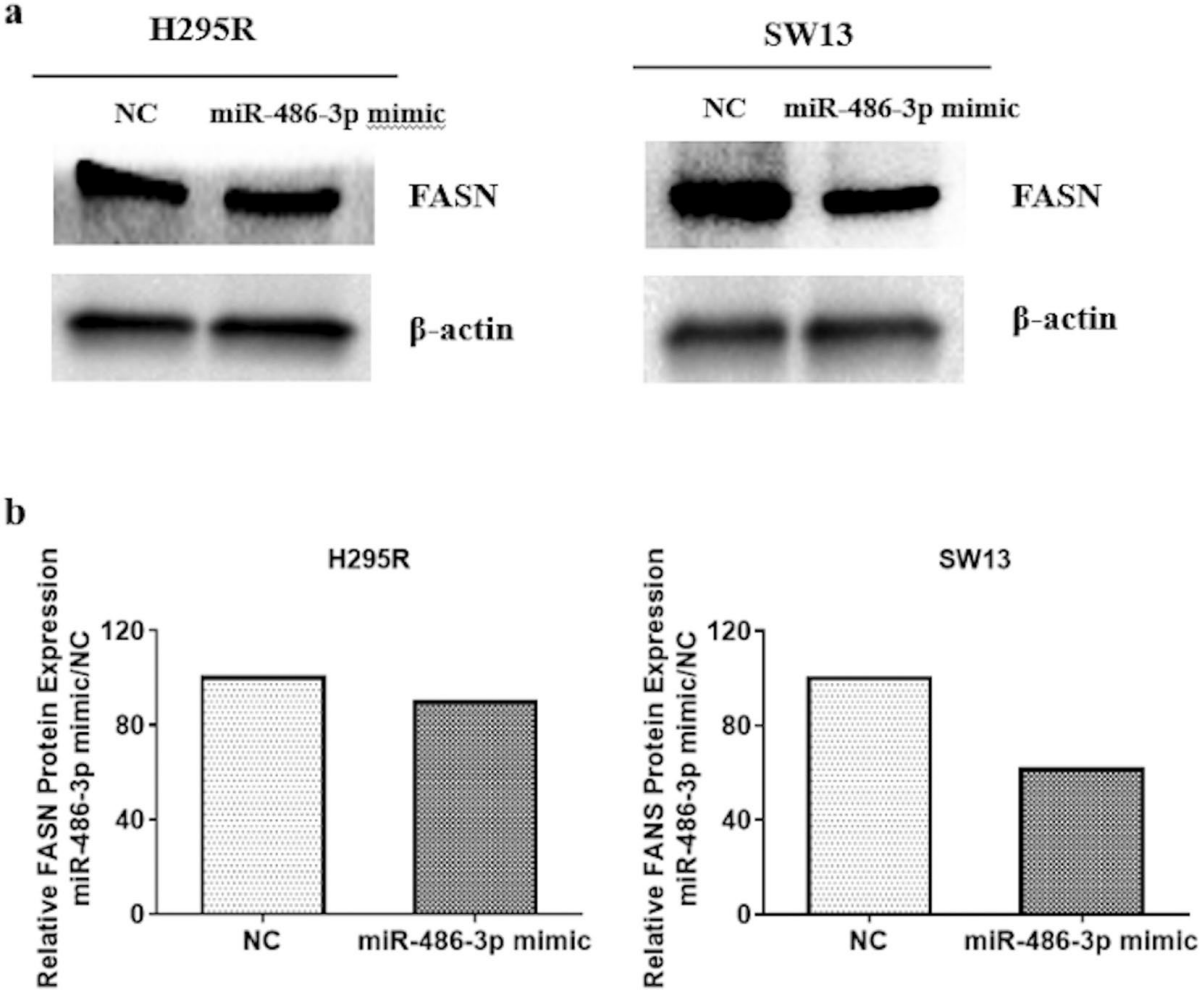
Protein assessments. (**a**) Western blot showing expression of FASN in H295R and SW13 cells transfected with miR-486-3p mimic compared to scrambled miRNA mimic (NC). (**b**) Graphical presentation of western blots analyses using β-actin for normalization. The experiments were performed three times. Full-length blots are presented in Supplementary Fig. 1.

H295R and SW13 cells were transfected with miR-486-3p mimic and miR-NC for 48 h and QRT-PCR analysis was performed to detect potential differential regulation of the target genes *ALDH2, FASN, GDI1, HINT1, KCND3 and MDGA1* in comparison to their expression level in control cells. Among these, *FASN* was the only gene showing a significant downregulation in the miR-486-3p overexpressing adrenocortical cell lines (Figs. 5 and 6). Furthermore, a reduced level of FASN protein expression could be visualized and seemed lower in miR-486-3p mimic transfected cells compared to corresponding miR-NC cells. This finding was most prominent in SW13 cells (Fig. 7).

### Overexpression of miR-486-3p inhibited palmitic acid synthesis

**Figure 8:**
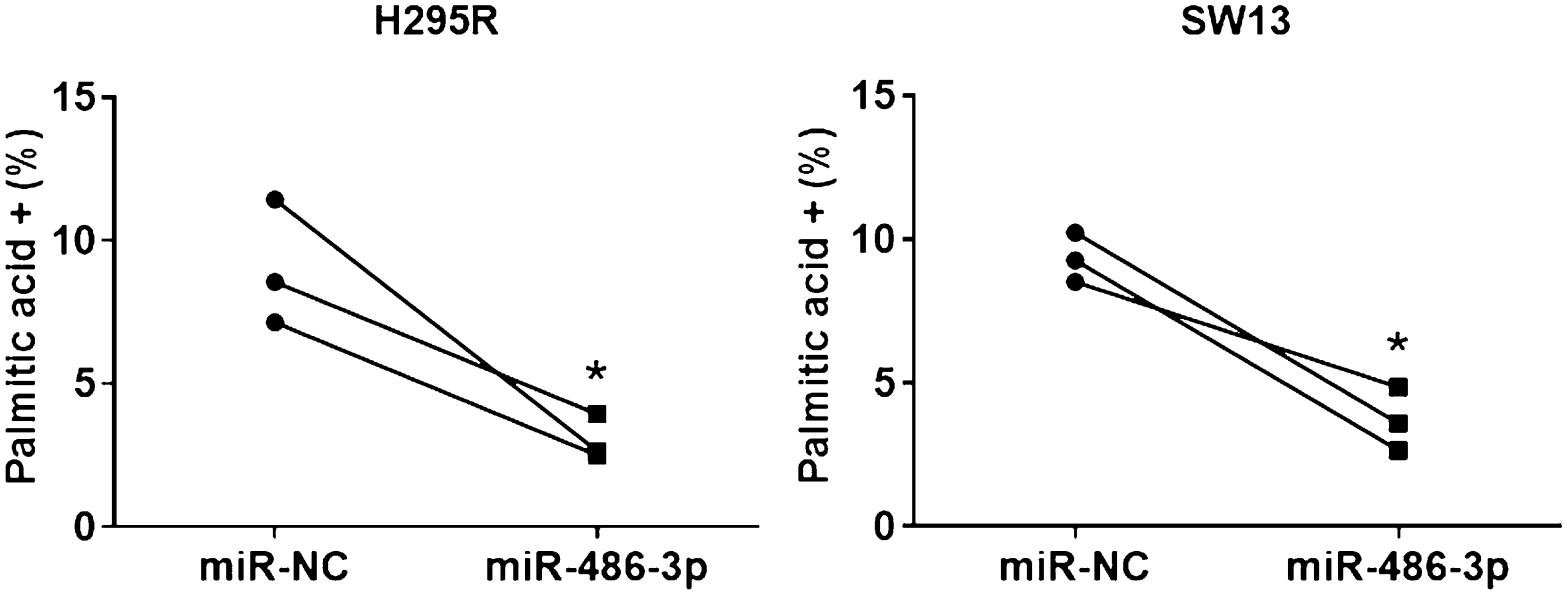
Palmitic acid detection by using flow cytometry assay. Palmitic acid levels were significantly (< 0.05) inhibited in both H295R as well as SW13 cells transfected with miR-486-3p mimic compared to corresponding cells transfected with miR-NC. The experiments were performed three times.

To further understand how the increased levels of miR-486-3p followed by decreased expression of FASN may impact adrenocortical proliferation we set out to analyze the downstream product of FASN activity, palmitic acid. Palmitic acid synthesis was significantly reduced, both in H295R and SW13 cells expressing miR-486-3p compared to miR-NC cells (*p* < 0.05) (Fig. 8).

## Discussion

Over the past decade emerging evidence strongly indicate that miRNAs play important roles in virtually every biological process and many human diseases. Notably, all types of neoplasms analyzed so far, including benign tumors, reveal miRNA alterations. Dysregulation of miRNAs influences all stages of transformation, from tumor initiation to dissemination, by regulating not only protein translation but also transcription and post-transcription processes^25^, thus they impact both nuclear and cytoplasmatic activity. Many miRNAs have oncogenic effects and are overexpressed in cancer^26,27^, whereas others that show suppressor capabilities may be underexpressed or deleted, also in human adrenocortical carcinoma^28–34^. They can be released to the circulation, and since miRNAs are remarkably stable, also in blood, they have the potential to function as clinical biomarkers^35,36^.

In the present study, we aimed at identifying miRNAs that might be of importance for initiation of tumorigenesis in adrenocortical cells and thus chose to study adrenals from a *Men1* knock-out mouse model showing few early signs of transformation. We chose to use a conventional knock-out mouse model, instead of a conditional, since the former mice have the same genotype as MEN1 patients, *i.e.* germ line heterozygous inactivation and preserved menin production from the retained wild type allele in the vast majority of adrenocortical lesions^2,37^. Since the goal was to recognize early molecular alterations more or less solely dependent on constitutionally low *Men1* gene dosage (heterozygous mice), we chose to compare the miRNA array profile of the adrenals of 10 months old *Men1*^+/−^ mice to that of the adrenals of equally young *Men1*^+/+^ animals. The *Men1*^+/−^ adrenals were not significantly larger than those of the wild type of the same strain, and they all expressed menin (Fig. 1). We deliberately avoided to use older mice with long-standing more prominent adrenal enlargements that might have acquired a multitude of additional mutations not necessarily representing a direct result of *Men1* being a haploinsufficient suppressor.

Altogether 31 miRNAs were identified as significantly differentially expressed in *Men1*^+/−^ mice compared to *Men1*^+/+^. The ratios of individual miRNAs between the genotypes were not particularly high, mean 42.3%, (range 20–106%), but the sample size, ten of each genotype, was obviously enough to find a fair number of significant alterations in such early proliferations. The majority of these 31 miRNAs were not earlier known as to be involved in tumor development, and the actual biological relevance of these finding warrants further studies. However, seven of the dysregulated miRNAs in *Men1*^+/−^ adrenals were already suggested to play active roles in tumorigenesis of different cancer types. The oncogenic miRNAs, miR-132-3p and miR-494-3p were significantly enhanced in *Men1*^+/−^ adrenals. These two are known to participate in regulation of transcription and proliferation in pancreatic cancer and glioma^26,27^. A somewhat intriguing finding was the differential expression of the suppressor miR-15 family; miR-497-5p and miR-195a-5p were upregulated in proliferating *Men1*^+/−^ adrenals. These miRNAs are known to be downregulated in human adrenocortical carcinomas compared to benign adenomas^16^. On the other hand, in the same study the miRNA profile of benign adenomas was also compared to that of normal adrenal cortices, and the latter comparison showed results in concordance with ours; miR-497-5p and -195a-5p were upregulated in benign adenomas compared to normal adrenals^16^.

We also found that three earlier described suppressor miRNAs (miR-214-5p, miR-330-5p and miR-486-3p) were significantly downregulated in *Men1*^+/−^ adrenals. MiR-214-5p has been describes as a key hub in control of cancer networks and is frequently differentially regulated^38^, but it is also reported to be downregulated in childhood as well as adult adrenocortical carcinomas^29–31,34^. MiR-330-5p has revealed antiproliferative effects in colorectal cancer^32^ and the tumor suppressor effect might be conducted via the mTOR/Akt-pathway^28^. Interestingly, the MEN1 protein itself has been suggested to be a negative regulator of AKT kinase activity^39^. The suppressor miR-486-3p has previously been studied in several tumor types; it regulated tumor progression in gastric cancer^40^ and revealed anti-proliferative effects in breast cancer^33^. Moreover, miR-486-3p was found to be underexpressed in childhood adrenocortical tumors^30^. In the present study, miR-486-3p held the highest rank by means of level of differential expression being downregulated by 60% compared to *Men1*^+/+^ adrenals.

One of many targets for miR-486-3p is the *FASN* gene. Accumulating evidence show that the enzyme FASN has huge impact on fatty acid synthesis pathways and links these processes to glucose metabolism^41–43^. The main function of FASN is to catalyze the synthesis of long chain saturated fatty acids, e.g. palmitate, from acetyl-CoA and malonyl-CoA^44^. Moreover, increased levels of FASN in cancer cells has been correlated to poor prognosis, and inhibition of FASN results in apoptosis of cancer cells^45–47^. We hypothesize that downregulation of miR-486-3p may be a key event in early adrenal tumorigenesis and a result of the *MEN1* heterozygosity per se. Low levels of miR-486-3p might lead to increased levels of FASN and thus increased supply of lipids which in turn is a prerequisite for membrane biogenesis in proliferating cells. It would indeed have been interesting to test this hypothesis by transfecting miR486-3p to human MEN1 adrenocortical cells, but this is of course not feasible. However, the hypothesis was somewhat strengthened by our finding upon transient transfection of miR-486-3p in human adrenocortical carcinoma cell lines H295R and SW13. Over-expression of miR-486-3p in these cells resulted in decreased proliferation as well as downregulation of FASN expression and subsequent decreased palmitate production.

We conclude that heterozygous inactivation of *Men1* in mouse adrenal tissue induces a specific miRNA profile, potentially regulating expression of target genes with effect on cellular processes in tumorigenesis, including transcription, nucleic acid metabolism, and lipid metabolism. We suggest that low levels of miR-486-3p might be an important early event in adrenocortical tumorigenesis and contributes to transformation by increasing the levels of FASN which in turn increases fatty acid production. Advanced adrenocortical carcinoma is a devastating disease with limited therapeutic options; FASN as a potential druggable target warrants further studies.

## Materials and methods

### Animals

The conventional germline heterozygous *Men1* mouse model was a kind gift by Professor Hayward of the Queensland Institute of Medical Research, Herston, Australia^5^. The phenotype of this strain mimics the human MEN 1 trait, *i.e.* development of macroscopically detectable classical *Men1* target lesions in the young adult mouse, from about nine months of age, followed by onset of proliferation of the adrenal glands. Experiments and animal maintenance were approved by and performed according to the guidelines and regulations of the local committees for animal care at Uppsala University (Permit Number: C187/14).

Since we aimed at recognizing early effects of *Men1* heterozygosity in the context of adrenal transformation, we chose ten months old animals to be compared to *Men1*^+*/*+^ of the same age and strain. Ten of each genotype were analyzed; we used eight male and two female *Men1*^+/−^, as well as six male and four female *Men1*^+*/*+^ mice. Size of the dissected adrenal was assessed as the product of length of the adrenal body long-axis and maximum perpendicular width. As suspected, size of the adrenals varied between individuals and some of the *Men*^+/−^ had slightly enlarged glands although the difference between genotypes was not significant; 8.6 ± 0.9 (SD) mm^2^ in *Men1*^+/−^ compared to 6.9 ± 0.7 (SD) mm^2^ in *Men1*^+/+^. Normal morphology and menin immunoreactivity (Fig. 1) were maintained in all specimens of both genotypes, also in the largest samples of *Men1*^+/−^ adrenal glands.

### Immunohistochemistry

Sections of mouse adrenals were deparaffinized and rehydrated in alcohol and then heat-retrived in Tris–EDTA, pH 9.0. Endogenous peroxidase activity was blocked by incubating the slides with Peroxidazed (Histolab Products AB, Askim, Sweden). After using Background Sniper (Histolab) to block nonspecific staining, the primary antibody Rabbit anti-menin (Bethyl Laboratories, Texas USA) was applied in dilution 1:800. Incubation with secondary antibody Rabbit-on-Rodent HRP Polymer (Histolab) was followed by chromogen Betazoid DAB staining. The sections were counterstained with hematoxylin.

### MiRNA preparation and miRNA array profiling

The dissected adrenals were immediately treated with RNA*later* RNA Stabilization Reagent (QIAGEN, Hilden, Germany) in order to protect the RNA in samples. The miRNeasy Micro Kit (QIAGEN) was utilized for purification of total RNA, including miRNA from the samples. Five hundred nanogram of total RNA from each sample were used to prepare biotinylated RNA according to the FlashTag Biotin HSR RNA labeling kit (P/N 703095 Rev. 2). One hundred twenty microliters of each sample were loaded to the Affymetrix miRNA 4.1 Array Plates. Finally, the arrays were hybridized, washed, stained and scanned with the GeneTitan Multi-Channel Instrument, all according to the GeneTitan Instrument User Guide for Expression Arrays Plates (Affymetrix, Santa Clare, California, USA).

### MiRNA array data analysis

The raw data was normalized in the free software Expression Console, provided by Affymetrix (http://www.affymetrix.com), using the robust multi-array average method first suggested by Li and Wong in 2001^48^. Subsequent analysis of data set, containing only the mouse probes, was carried out in the freely available statistical computing language R (http://www.r-project.org). In order to search for differentially expressed genes between *Men1*^+*/−*^ and *Men1*^+*/*+^ mice, an empirical Bayes moderated t-test was applied to employ the robust version of the lmFit function from the ‘limma’ package^49,50^. To address the problem with multiple testing, the p-values were adjusted using the method of Benjamini and Hochberg^51^.

### Prediction of miR-486-3p target genes

Target genes of miR-486-3p, as the top-ranked differentially expressed miRNA in *Men1*^+*/*^*-* adrenal, was further investigated. The prediction was performed by using TargetScan (http://www.targetscan.org/) free online software program along with findings from proteomics profiling of adrenals of the same *Men1* mouse model^24^. The biological functions of the potential miRNA targets were further investigated using Ensembl (http://www.ensembl.org/) gene ontology information.

### Human adrenocortical cancer cell lines

Two human adrenocortical carcinoma cell lines, H295R and SW13 cells (ATCC, Manassas, Virginia, USA), were used in this study. The cells were cultured at 37°C and 5% CO_2_-humidified atmosphere. H295R cells were cultured in Dulbecco’s modified Eagle’s medium/Ham F12 (DMEM/F12) medium, supplemented with 1% ITS Liquid Media Supplement, 100 units/ml of penicillin, 100 μg/ml streptomycin (1% PEST) and 2% Nu-serum. SW13 cells were cultured in DMEM/F12 medium, supplemented with 1% PEST and 10% fetal bovine serum. All reagents were purchased from Thermo Fischer Scientific (Waltham, Massachusetts, USA).

### MiRNA mimic transfection

MiR-486-3p mimic and scrambled miRNA mimic (Thermo Fisher Scientific) were used to transfect H295R and SW13 cells. The mirVana mimic is double strand oligonucleotides mimicking mature miRNA and scrambled miRNA mimic is used as non-targeting negative control (miR-NC). The transfection experiment was performed by the reverse transfection procedure, according to the manufacturer's instructions, using Lipofectamine RNAiMAX (Thermo Fisher Scientific). A master mix including 25 pmol of miR-486-3p mimic or scrambled miRNA mimic (Thermo Fisher Scientific), 7.5 µL of Lipofectamine RNAiMAX and 250 µL Opti-MEM Medium (Thermo Fisher Scientific) per well was added to six-well plates, and then 6 × 10^5^ H295R cells and 2 × 10^5^ SW13 cells were seeded in each well. Cells were incubated in a 5% CO_2_-humidified atmosphere incubator at 37 °C for 48 h. The transfection experiments were performed at least three times and used for gene and protein expression analysis, cell proliferation and palmitic acid assay.

### RNA extraction and quantitative real time PCR (QRT-PCR) analysis of miRNA and mRNA expression

Total RNA from cells of the adrenocortical carcinoma cell lines H295R and SW13 were isolated by using the mirVana miRNA isolation kit (Thermo Fisher Scientific) according to the manufacturer’s instructions. The purified RNA was eluted with nuclease-free water (Thermo Fisher Scientific) and was stored at -70ºC until further analysis. The RNA concentration was measured by using the NanoDrop 1000 (Thermo Fisher Scientific). One µg of total RNA from cell lines was reverse transcribed by the TaqMan MicroRNA Reverse Transcription Kit (Thermo Fisher Scientific) to detect miRNA expression. The primers used to analyze miRNA expression are described in the upper part of Supplementary Table 1. Moreover, 1 µg of total RNA from the cells were converted to cDNA with the iScript cDNA synthesis Kit (Bio-Rad, Hercules, California, USA) to detect gene expression and the primers are described in the lower part of the Supplementary Table 1. The QRT-PCR reaction was run on the Stratagene Mx3005P real-time PCR System (Agilent Technologies, Santa Clara, California, USA). Each QRT-PCR reaction of miRNA was carried out in 20 µL comprising 2 × TaqMan Universal PCR Master Mix II without UNG (Thermo Fisher Scientific), 20 × TaqMan Small RNA Assay and RT product. Each QRT-PCR reaction of mRNA was carried out in 20 µL including 2 × SsoAdvanced Universal SYBR Green Supermix (Bio-Rad), 500 nM concentrations of forward and reverse primers and 10 ng of cDNA. The reaction mixture was incubated at 95ºC for 10 min, followed by 40 cycles at 94ºC for 15 s and 60ºC for 1 min. The data were evaluated by the 2^−∆∆CT^ method^52^ using either miRNA level of miR-16 or mRNA level of *β-actin* as internal control. All QRT-PCR expression analyses were performed at least three times.

### Western blot analysis

Whole-cell protein lysates of H295R and SW13 cells were extracted by using radio-immunoprecipitation assay buffer (Sigma-Aldrich, St. Louis, Missouri, USA). Protein concentrations were determined using Coomassie Plus Better BradFord Assay (Thermo Fisher Scientific). Aliquots of 20 µg protein lysates were resolved by precast 4–20% Mini-PROTEAN TGX gels (Bio-Rad) and then transferred to 0.2-µm nitrocellulose membranes (Cell Signaling Technology, Danvers, Massachusetts, USA). PageRuler pre-stained protein ladder (Thermo Fisher Scientific) was used for the apparent size of proteins. The membranes were blocked with 5% milk in Tris-buffered saline solution containing 0.1% Tween-20 and then blotted with the primary antibody overnight at 4˚C. After washing, the membranes were incubated with horseradish peroxidase-conjugated anti-rabbit or anti-mouse IgG antibodies (GE Healthcare, Chicago, Illinois, USA), and proteins were visualized using ECL Plus Western Blotting Detection Systems (GE Healthcare) on a cooled charge-coupled device camera (Bio-Rad). Densitometrical analysis of the immunoblots was performed and quantified using the Imagelab software (Bio-Rad). Antibodies against Fatty acid synthase (#3180) and β-actin (#4970) were purchased from Cell Signaling Technology. Western blot analyses were performed three times.

### Flow cytometry analysis

Click-IT Plus EdU Alexa Fluor 647 Flow Cytometry Assay Kit (Thermo Fisher Scientific) was used to analysis cell proliferation in miRNA mimic transfected adrenocortical carcinoma cell lines H295R and SW13. In brief, the transfected cells were incubated with 10 µM EdU for two hours and the cells were then harvested for flow cytometry analysis. In addition, Click-IT palmitic acid azide (Thermo Fisher Scientific) was used to detect palmitic acid in miRNA mimic transfected H295R and SW13 cells. Briefly, transfected cells were incubated overnight in 25 mM palmitic acid azide and the cells then collected for flow cytometry analysis. The experiments were performed three times.

### Statistical analysis

The statistical significance of the difference between two groups was evaluated by paired t test using GraphPad Prism 6 (San Diego, California, USA); *p* value < 0.05 was considered significant.

## Supplementary Information

Supplementary Information.
