# Supplementary material for: MiR-486-3p was downregulated at microRNA profiling of adrenals of multiple endocrine neoplasia type 1 mice, and inhibited human adrenocortical carcinoma cell lines
Source: Sci Rep. 2021 Jul 20;11:14772. doi: 10.1038/s41598-021-94154-z (PMC8292366; doi:10.1038/s41598-021-94154-z)
Supplement: Supplementary file 1 — Supplementary Information. [file 41598_2021_94154_MOESM1_ESM.docx]

**Supplementary Table 1. Primer pairs used for Q-RT-PCR analysis**

| **Name** | **Sequence** | **Accession number** |
| --- | --- | --- |
| hsa-miR-16 | F: 5'-TAGCAGCACGTAAATATTGGCG-3' | MI0000070 |
| hsa-miR-486-3p | F: 5'-CGGGGCAGCUCAGUACAGGAU-3' | MI0002470 |

| **Symbol** | **Description** | **Primer Sequences** | **Product (bp)** |
| --- | --- | --- | --- |
| *ALDH2* | aldehyde dehydrogenase 2 | F: 5'-GAGAGTGACCTTGGAGCTGGGGG-3' | 178 |
|  |  | R: 5'-CTCCGCTCCACAAACTCATCATA-3' |  |
| *BACT* | Beta actin | F: 5'-AAGGAGATCACTGCCCTGGC-3' | 131 |
|  |  | R: 5'- CCACATCTGCTGGAAGGTGG-3' |  |
| *FASN* | fatty acid synthase | F: 5'- TACGTACTGGCCTACACCCAGA -3' | 100 |
|  |  | R: 5'- TGAACTGCTGCACGAAGAAGCATAT -3' |  |
| *GDI1* | GDP dissociation inhibitor 1 | F: 5'- CGCCAACTCCTGCCAAATAAT -3' | 91 |
|  |  | R: 5'- CACGTTGTGTGCATAGGAGATCA -3' |  |
| *HINT1* | histidine triad nucleotide binding protein 1 | F: 5'- ATGACATTTCCCCTCAAGCA -3' | 179 |
|  |  | R: 5'- ACCTTCATTCACCACCATTC -3' |  |
| *KCND3* | potassium voltage-gated channel subfamily D member 3 | F: 5'- ATAAACGCAGGGCACAAAAG -3' | 178 |
|  |  | R: 5'- AGGTGATGATGCTGGCTCTC -3' |  |
| *MDGA1* | \|  \| MAM domain containing glycosylphosphatidylinositol \| \| --- \| --- \| | F: 5'- GAAGCAGACGGATCCCAATA -3' | 192 |
|  |  | R: 5'- GGGGGTCAGTCTTTGGTACA -3' |  |

**
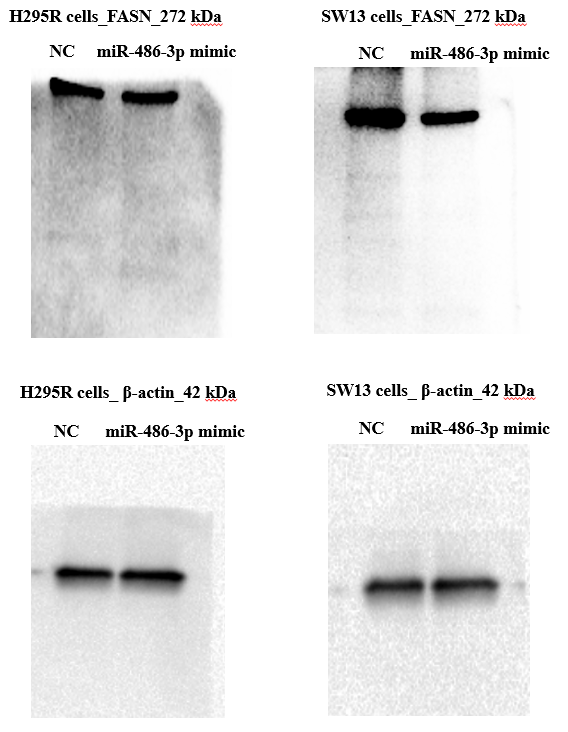
**

**Supplementary Figure 1.** Original Western blot analysis with full-length blots of figure 7.
